# Analysis of self-heating of thermally assisted spin-transfer torque magnetic random access memory

**DOI:** 10.3762/bjnano.7.160

**Published:** 2016-11-11

**Authors:** Austin Deschenes, Sadid Muneer, Mustafa Akbulut, Ali Gokirmak, Helena Silva

**Affiliations:** 1 Electrical Engineering, University of Connecticut, Storrs, Connecticut, 06082, USA

**Keywords:** FEM modeling, Joule heating, self-heating, spin torque transfer magnetic random access memory (STT-MRAM), thermoelectrics

## Abstract

Thermal assistance has been shown to significantly reduce the required operation power for spin torque transfer magnetic random access memory (STT-MRAM). Proposed heating methods include modified material stack compositions that result in increased self-heating or external heat sources. In this work we analyze the self-heating process of a standard perpendicular magnetic anisotropy STT-MRAM device through numerical simulations in order to understand the relative contributions of Joule, thermoelectric Peltier and Thomson, and tunneling junction heating. A 2D rotationally symmetric numerical model is used to solve the coupled electro-thermal equations including thermoelectric effects and heat absorbed or released at the tunneling junction. We compare self-heating for different common passivation materials, positive and negative electrical current polarity, and different device thermal anchoring and boundaries resistance configurations. The variations considered are found to result in significant differences in maximum temperatures reached. Average increases of 3 K, 10 K, and 100 K for different passivation materials, positive and negative polarity, and different thermal anchoring configurations, respectively, are observed. The highest temperatures, up to 424 K, are obtained for silicon dioxide as the passivation material, positive polarity, and low thermal anchoring with thermal boundary resistance configurations. Interestingly it is also found that due to the tunneling heat, Peltier effect, device geometry, and numerous interfacial layers around the magnetic tunnel junction (MTJ), most of the heat is dissipated on the lower potential side of the magnetic junction. This asymmetry in heating, which has also been observed experimentally, is important as thermally assisted switching requires heating of the free layer specifically and this will be significantly different for the two polarity operations, set and reset.

## Introduction

Spin torque transfer magnetic random access memory (STT-MRAM), a type of non-volatile memory, functions through the resistance ratio between the parallel (ON) and anti-parallel (OFF) states of two ferromagnetic domains on either side of a thin insulating barrier. These ferromagnetic domains and the insulating barrier make up the magnetic tunnel junction (MTJ) of the STT-MRAM device. The device is switched between the ON and OFF states by passing a current of the appropriate direction through the MTJ allowing the rotational spin of tunneling carriers to “torque” the free magnetic domain into the desired alignment. STT-MRAM exhibits desirable endurance, speed, and scaling properties. The write process of STT-MRAM has been shown to be highly reactive to thermal processes [1–3]. Bi et al. showed that utilizing external heat sources to elevate the temperature of the device by 50 K could reduce power consumption by up to 4.8% [2]. Bandiera et al. showed practical operation of STT-MRAM devices below the 22 nm node by enhanced self-heating, up to temperatures of 360 K to 523 K, through manipulation of the material stack [3]. However, changing the material stack compromises the desired anisotropy levels and external heat sources impact device density. Thus, these methods for thermal assistance can be considered limited in their potential.

**Figure 1:**
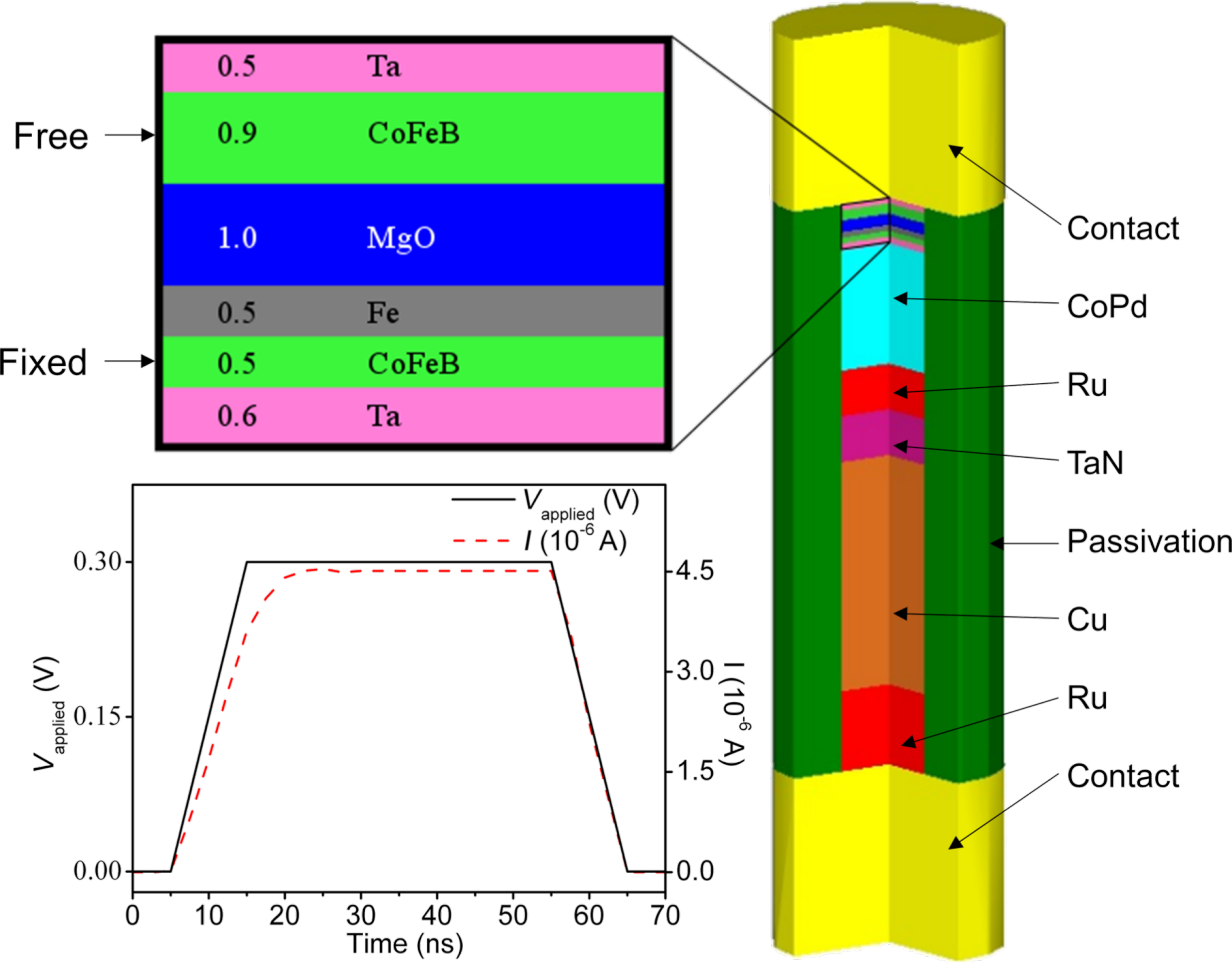
MRAM device structure with a stack radius of 10 nm. Materials, with exception of contact regions, are proportional to actual device model. The CoFeB–MgO–Fe–CoFeB MTJ is shown in the enlarged image, where the dimensions are given in nanometers. The inset shows the applied voltage and resultant current waveform for 0.3 V pulse.

In this work we perform an electro-thermal analysis of the self-heating process for a standard perpendicular magnetic anisotropy STT-MRAM device (Figure 1) to understand the relative contributions of the different heat mechanisms involved and the effect of external device parameters such as passivation material, current polarity and contact configurations, and to determine how such design choices may enhance thermal assistance for STT-MRAM.

## Computational details

### Model

We have modeled the electro-thermal behavior of the device using the “COMSOL Multiphysics Finite-Element Modeling” software. Voltage, current, and temperature are obtained by solving the coupled current (Equation 1) and heat (Equation 2) equations:

[1]
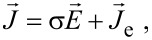


**Figure 2:**
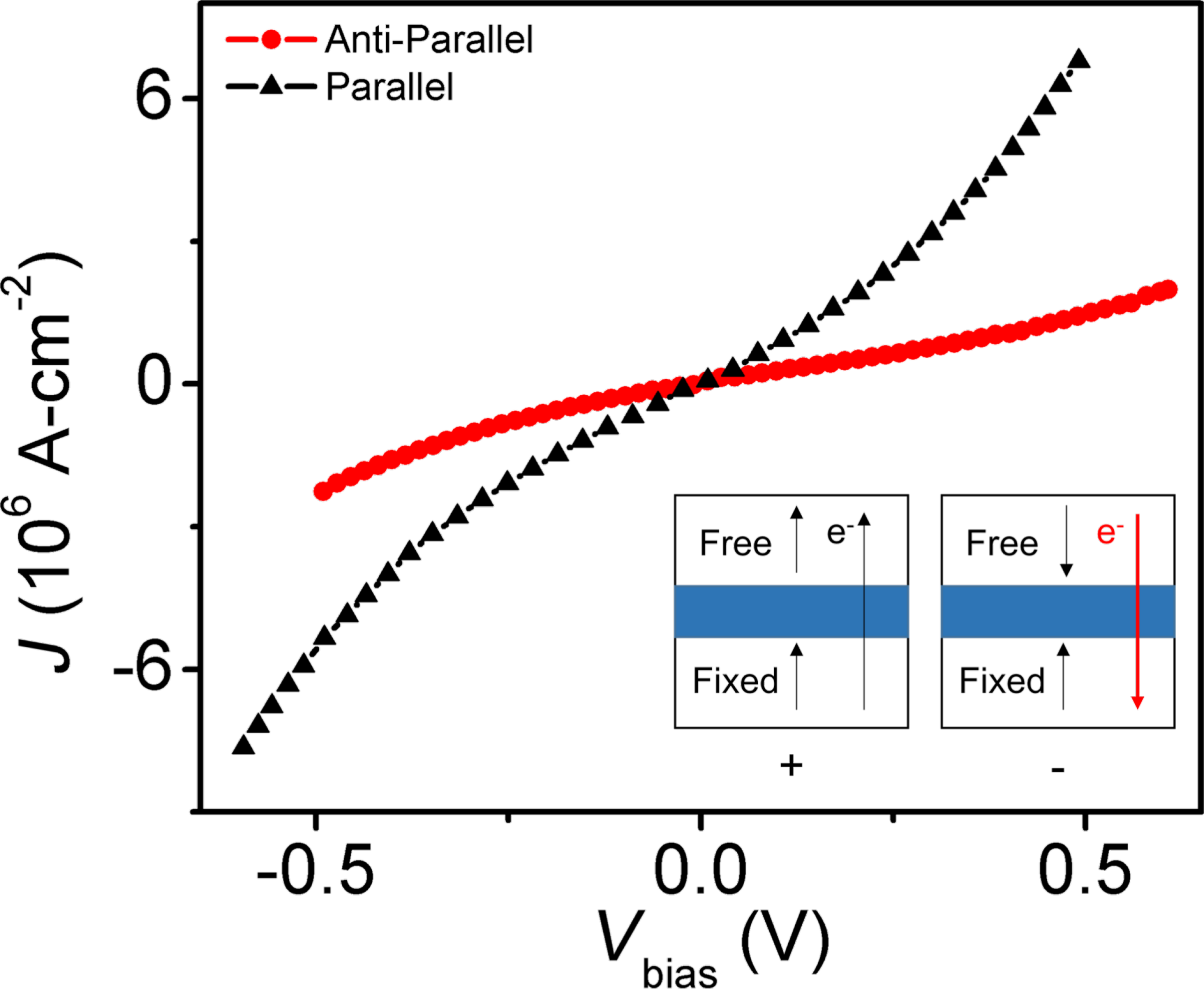
Characteristic *J*–*V* curve built from literature [4–7] with similar CoFeB/MgO/CoFeB MTJ with thickness of the order of a few nanometers. Probes are used to find bias voltage across the tunnel junction. Curves are extrapolated via nearest-function method for 0.6 V simulations. Insets show the flow of carriers, and implied material properties, for a given initial free and fixed magnetic layer alignment. Simulations with initially parallel (ON) free/fixed layers use parallel properties, whereas simulations with initially anti-parallel (OFF) free/fixed layers use anti-parallel properties.

[2]
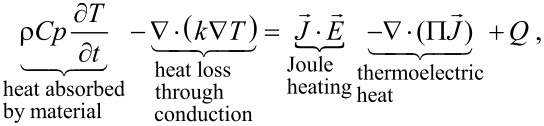


where 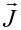 is the current density, σ is the electrical conductivity, 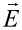 is the electric field, 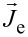 is the external current source used to model the tunnel junction, ρ is the mass density, *C_p_* is the heat capacity, *T* is the temperature, *t* is the time, *k* is the thermal conductivity, and Π is the net Peltier coefficient. Thermoelectric Peltier, and Thomson heat terms are included in the heat equation (Equation 2). Tunneling through the device is modeled using an external circuit that circumvents the thin insulating barrier with the resistance being characterized by experimental *J*–*V* curves obtained for the same junction from literature [4–7] (Figure 2).

This external circuit to model the tunnel junction is the only contribution of 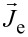
and requires the corresponding heat contributions to be added separately as external heat sources. Heat released or absorbed at the tunneling junction (*Q_t_*) is modeled using the probabilistic equation for hot tunneling carrier relaxation [1]:

[3]
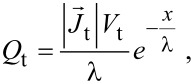


where 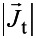 is the magnitude of tunneling current density, *V*_t_ is the electric potential drop across the junction, λ is the inelastic scattering mean free path, and *x* is the stack position. The Peltier heat associated with the tunneling electrons is modeled using:

[4]
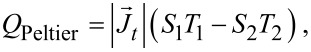


where 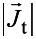 is the tunneling current density, and *S* and *T* are the Peltier coefficients (*S* is the Seebeck coefficient, *T* is the temperature) on either side of the junction. The heat contributions from Equation 3 and Equation 4 are released or absorbed on the appropriate side of the tunneling barrier depending on the current polarity.

The MRAM device modeled (Figure 1) is composed of the following layers: contact | 0.5 Ta | 0.9 CoFeB (free) | 1 MgO | 0.5 Fe | 0.5 CoFeB (fixed) | 0.6 Ta | [0.25 Pd | 0.8 Co] × 10 | 4 Ru | 4 TaN | 20 Cu | 7 Ru | contact, where the numbers before the alloy composition of each layer represent the thickness of that layer in nanometers. The radius of the cylindrical material stack is 10 nm. The passivation layer surrounding the stack is 5 nm thick. In the material stack the CoFeB layers are the ferromagnetic domains of the MTJ, MgO is the thin insulating layer, and Ta/CoPd/Ru is the synthetic antiferromagnetic layer. The material stack modeled is from a device reported by Worledge et al. [8]. The thermal stability factor of the free ferromagnetic layer of the device, derived from Equation 5 using materials properties from [8–9], is 38.7 *k*_B_T:

[5]
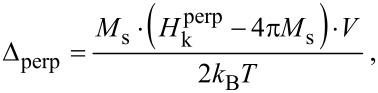


where *Δ*_perp_ is the perpendicular thermal stability factor, *M*_s_ is the saturation magnetization, 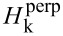 is the perpendicular effective anisotropy field, *V* is the volume of the free layer, *k*_B_ is the Boltzmann constant, and *T* is the local temperature.

**Figure 3:**
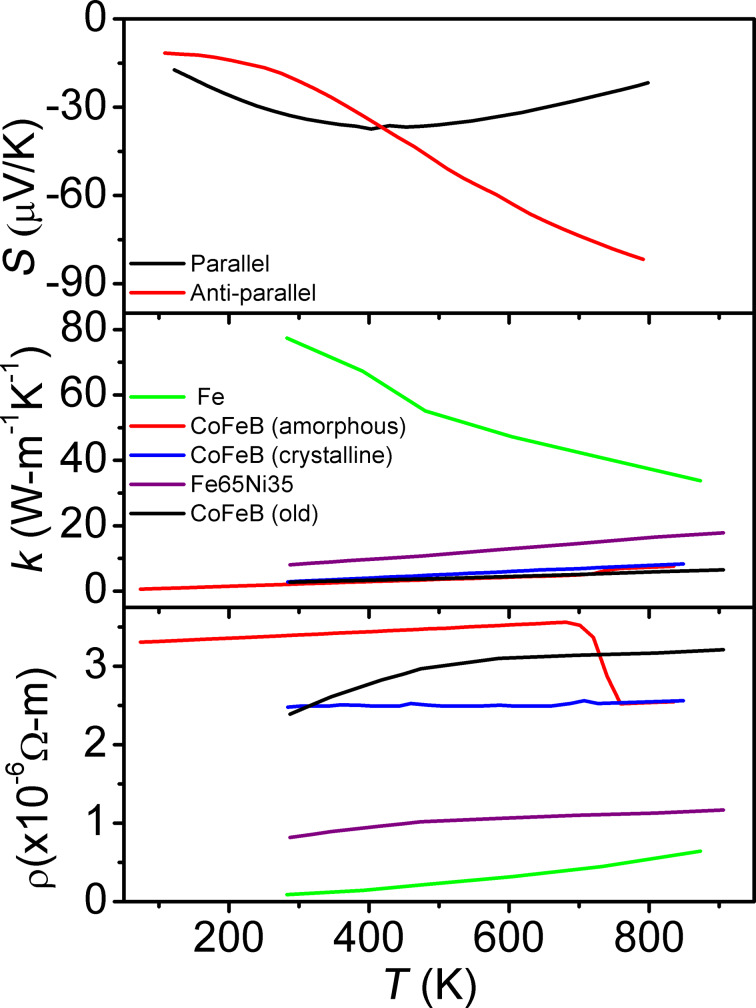
Graph of materials properties used in model construction. From top to bottom: Seebeck values of CoFeB for parallel (ON) and anti-parallel (OFF) states [11], thermal conductivity of materials [8,10], electrical resistivity of materials [8]. Crystalline and amorphous CoFeB values came from literature [12].

Temperature-dependent materials properties [7,10–14] (Figure 3) are used for CoFeB, MgO, and Fe. The temperature-dependent thermal conductivity of CoFeB is calculated using the Wiedemann–Franz law:

[6]
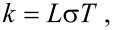


where *L* is the Lorenz number. It is assumed the annealing temperature was sufficiently low so as to not cause crystallization [7] on the MgO–CoFeB interface, leaving the CoFeB completely amorphous. The Pd–Co layers are treated as an alloy due to the large number of sub-nanometer thick layers. The temperature-dependent materials properties of the PdCo alloy were derived using the behavior of similar alloys [10] transposed onto point data [15]. The material properties of the various common passivation materials considered [16] (Table 1) are from the element library of COMSOL for SiO_2_ and Si_3_N_4_, and from literature for low-temperature plasma-enhanced chemical vapor deposition SiO_2_ [2,17] and Si_3_N_4_ [18]. The “ON” and “OFF” states of the device are represented via interchangeable sets of materials properties for the CoFeB layers [4,13] (Figure 2, Figure 3) that are chosen based on the desired switching operation to be simulated (anti-parallel properties to simulate an OFF–ON switching operation, parallel properties for the converse operation, see inset in Figure 2).

**Table 1:** Properties of passivation materials.^a^

material	relative permittivity	thermal conductivity (W/m·K)	heat capacity (J/kg·K)

Si_3_N_4_*^b^	7	30	170
Si_3_N_4_	7.5	30	700
SiO_2_*	5	1.1	650
SiO_2_	3.9	1.4	730

^a^Properties at room temperature are given. Values are from the “COMSOL” element library for SiO_2_ and Si_3_N_4_, (high-temperature growth or deposition, considered here as a limit case) and from literature for SiO_2_* [1,15] and Si_3_N_4_* [16]. ^b^The asterisk indicates material properties for low-temperature plasma-enhanced chemical vapor deposition (PECVD) silicon dioxide or silicon nitride.

### Simulation procedures

**Figure 4:**
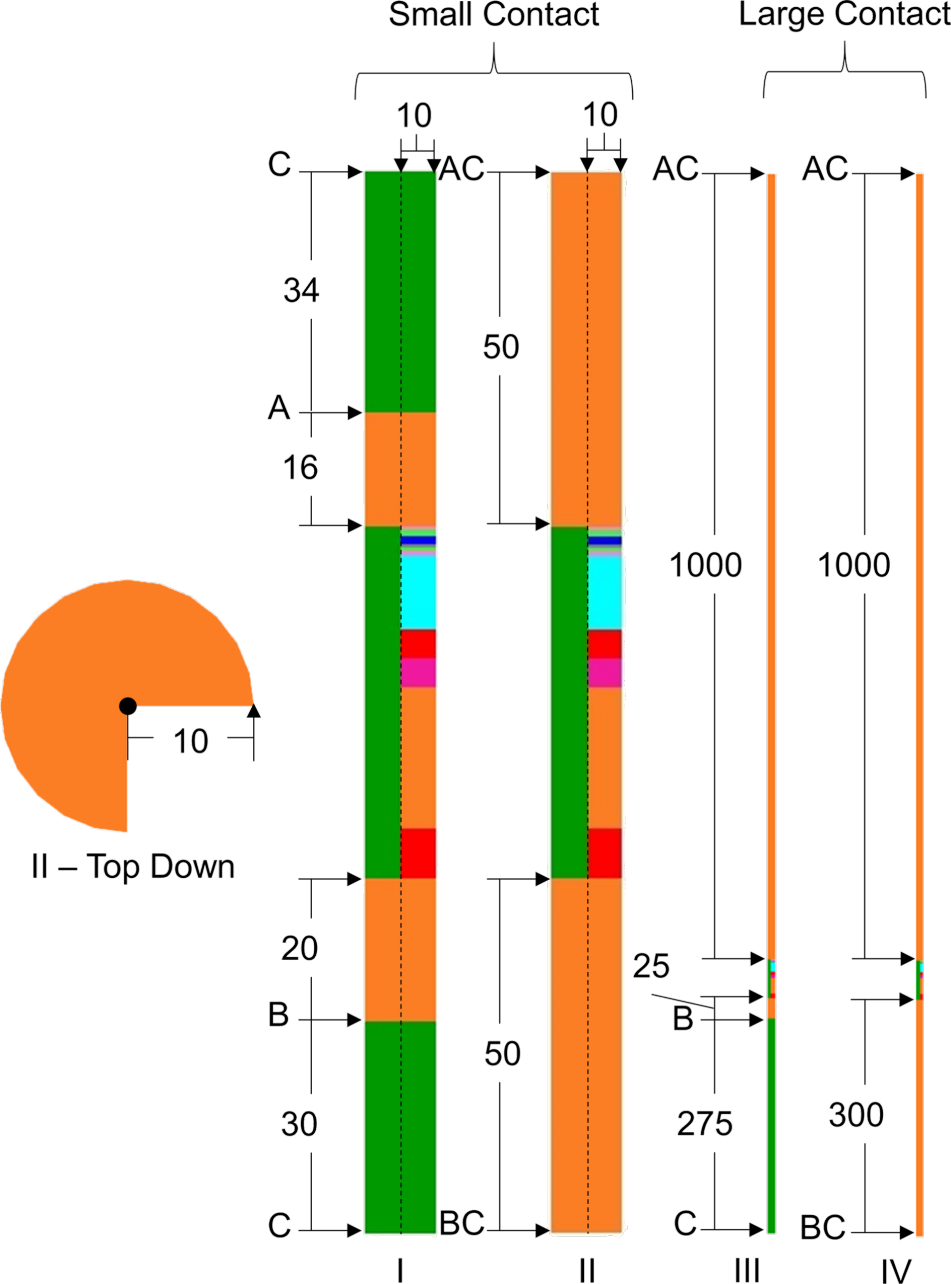
Schematic top-down view and quarter cross-section view of the simulated geometries. The dotted lines indicate the axis of symmetry for the 2D cylindrical simulations. The active area of the device is surrounded by an insulating passivation layer (green). Four contact configurations are considered to model different layers and interconnects in an actual array: small passivation–Cu (I), small Cu (II), large passivation–Cu (III), and large Cu (IV). A thermal boundary resistance between the metal and insulating layers (A and B interfaces) is considered in some of the simulations. Key: C = heat sink (*T* = 300 K), A = top contact, B = bottom contact, AC = heat sink and top contact, BC = heat sink and bottom contact. All dimensions shown are in units of nanometers. All stack configurations have an active magnetic tunnel junction radius of 10 nm. Different scale factors are used for I, II and III, IV to account for significant differences in device height.

Analysis of the self-heating of the device is performed for different passivation materials (Table 1), positive and negative current polarity, and four contact configurations (Figure 4), using voltage pulses as shown in the inset of Figure 1. The bias voltage dependence of the TMR is not considered and the resistance values of the MTJ in the ON and OFF states, derived from Figure 2, are assumed to be constant during the switching pulses. A positive voltage is applied at point A and ground at point B in Figure 4 (positive polarity, to simulate ON–OFF switching) or the converse (negative polarity, to simulate OFF–ON switching). The thermal boundary conditions, represented as C in Figure 4, are set to 300 K at all times. The peak temperatures within the free CoFeB layer, fixed CoFeB layer, and for the whole device are recorded for each pulse as *T*_free_, *T*_fixed_, and *T*_peak_, respectively.

The contact regions in Figure 1 are changed between sets of simulations in order to model the device in different thermal anchoring conditions. The four configurations used to model these contact regions are shown in Figure 4. To model a discrete STT-MRAM cell, the configurations I and II are simulated and referred to as “small contact” devices. To model a device in a memory array, the “large contact” configurations III and IV are used to emulate large thermal coupling [19]. To model different heat paths through metal and passivation layers, the configurations with passivation–Cu contacts (I, III) are simulated with and without thermal boundary resistances (TBR) applied on the passivation–Cu interfaces. This TBR is modeled as a 1 nm thick virtual layer with a thermal conductivity of 0.041 W/m·K.

Simulation sets are performed for configurations II and IV in positive polarity, with pulse amplitudes ranging from 0.2 to 0.4 V, with all passivation materials considered in Table 1. Additional simulations are run for SiO_2_ passivation layer, which exhibits the most favorable contributions to self-heating (see Results and Conclusion), on configurations I to IV with pulse amplitude ranging from 0.2 to 0.6 V for both positive and negative polarity to further analyze the effects of thermal anchoring and current polarity.

## Results and Discussion

**Figure 5:**
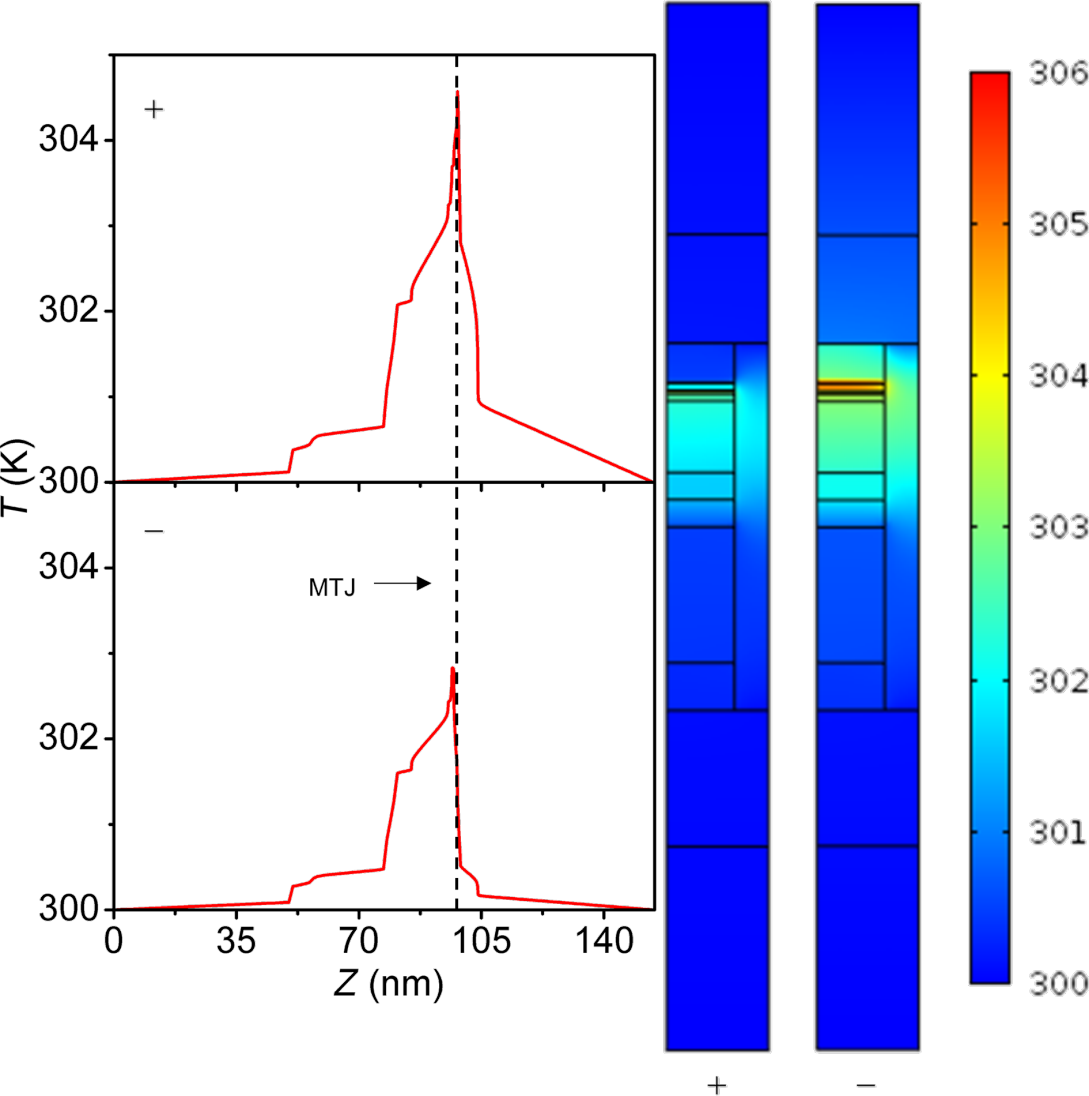
Thermal profiles of configuration II with SiO_2_ passivation with an applied voltage of 0.3 V in “positive” polarity (top) and “negative” polarity (bottom). Left figures show the temperature as a function of the vertical position with 0 nm being the bottom of the device. The dotted line in the plots represents the center of the MTJ. Note the asymmetry around the MTJ.

**Figure 6:**
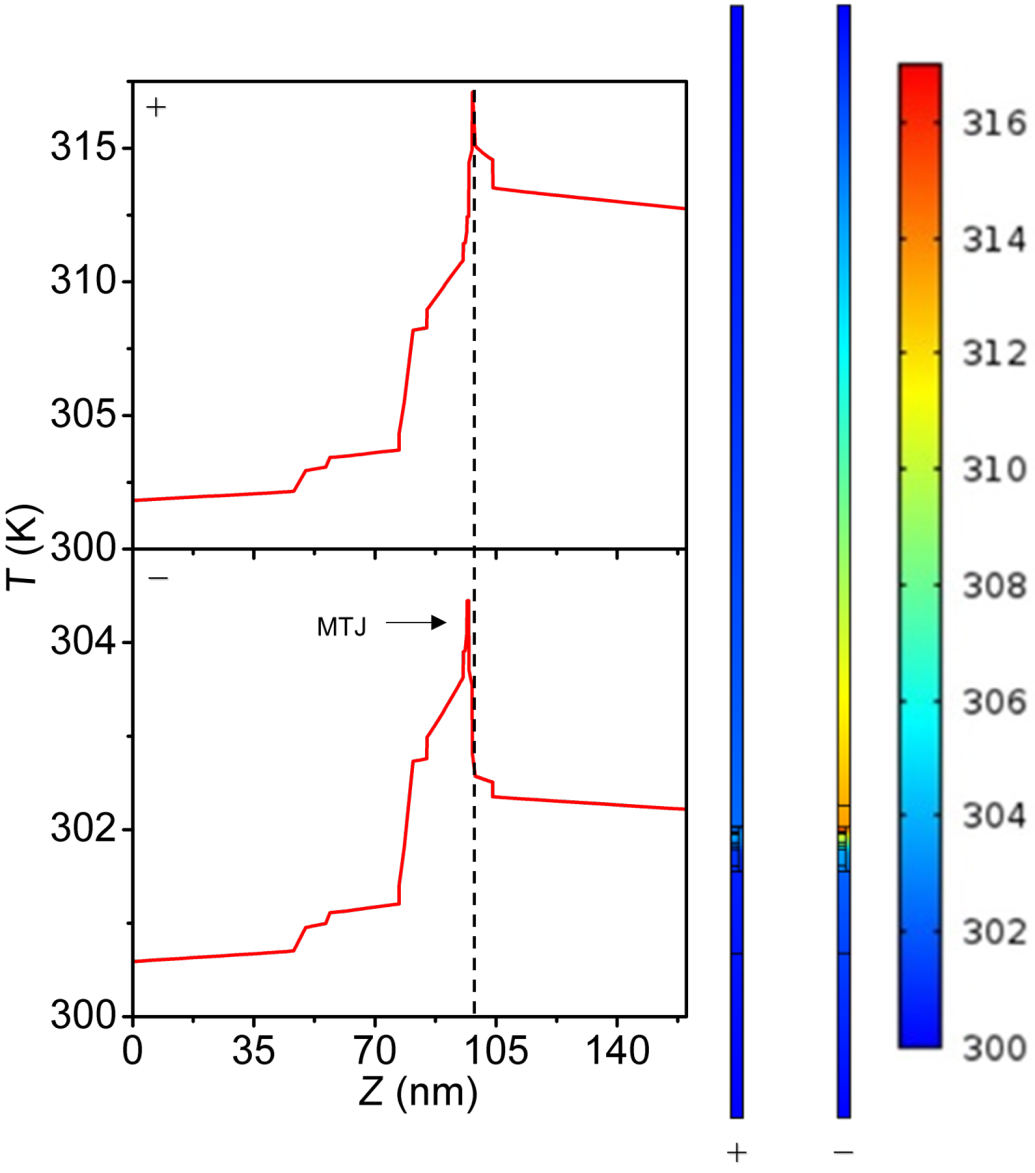
Thermal profiles of configuration IV with SiO_2_ passivation with an applied voltage of 0.3 V in “positive” polarity (top) and “negative” polarity (bottom). The left panels show the temperature as a function of the vertical position. The dotted line in the plots represents the center of the MTJ. Note the asymmetry around the MTJ. The free layer in the magnetic tunnel junction reaches ca. 316 K in positive polarity while it only reaches ca. 303 K in negative polarity.

**Figure 7:**
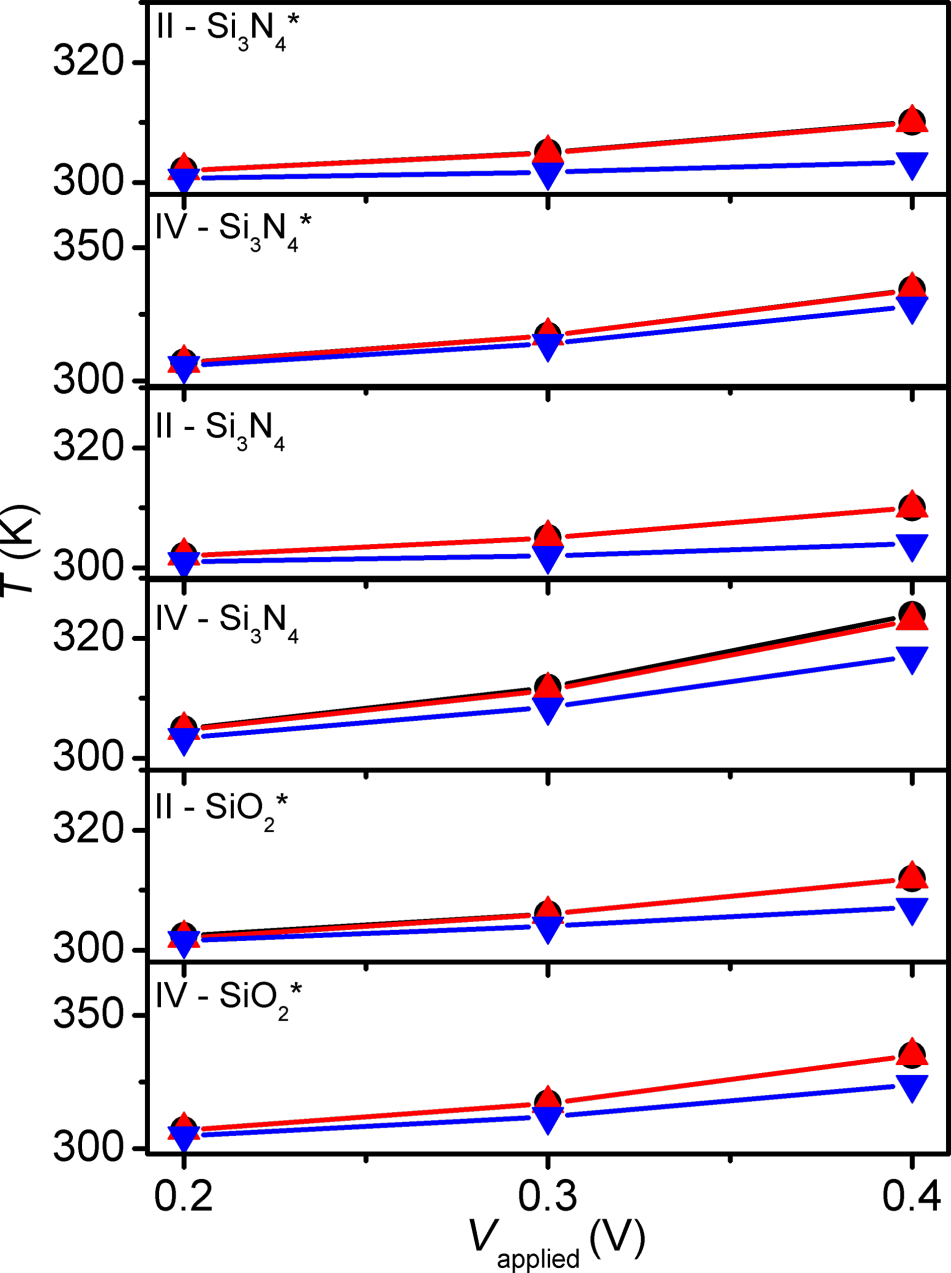
Results for different passivation materials. *T*_peak_ (black circles), *T*_free_ (red upward triangles) and *T*_fixed_ (blue downward triangles), recorded for configurations II and IV from 0.2 to 0.4 V in positive polarity.

**Figure 8:**
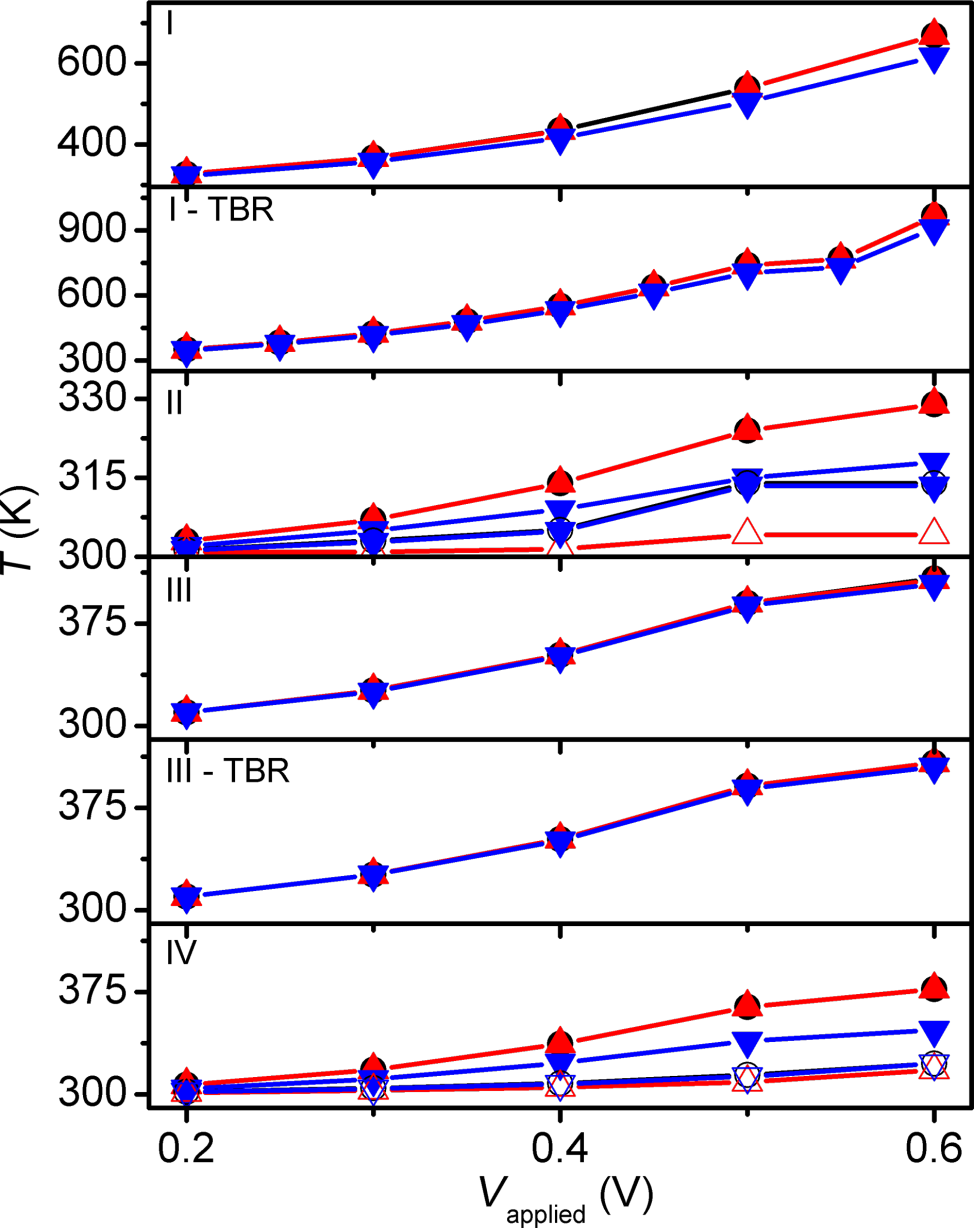
*T*_peak_ (black circles), *T*_free_ (red upward triangle) and *T*_fixed_ (blue downward triangles), recorded for large- and small-contact configurations. Simulations carried out for SiO_2_ passivation. Configurations II and IV feature positive polarity (solid symbols) and negative polarity (open symbols). Configurations I and III are simulated with and without thermal boundary resistance (TBR) at the passivation–Cu interfaces.

Asymmetry of heating around the MTJ, shown by the simulation results in Figures 5–8, is a result of the asymmetric heat contributions from *Q*_Peltier_ and *Q*_Tunneling_ and interfacial layers with high thermal resistance surrounding the thin insulating MgO layer. The asymmetry in heating increases with overall heating as shown below in Figure 7 and Figure 8 for configurations II and IV. Experimental observations of asymmetric heating in MRAM devices have been reported earlier by Gapihan et al. who predicted that the favorable current direction can result in a 10% reduction in the heating power density required for switching [20].

The temperature *T*_peak_ of the small-contact configurations at expected operational voltages of STT-MRAM (0.3 V) range from 305 K for configuration II in positive polarity with Si_3_N_4_ passivation, to 424 K for configuration I with TBR in positive polarity with SiO_2_ passivation.

Large-contact configurations exhibit higher asymmetric tendencies and, on average, have a higher *T*_peak_ by 11.8 K compared to observations for similar small-contact simulations (same passivation material, voltage applied, and polarity). This is the result of more pronounced geometric asymmetry and a larger heat capacity of the configurations III and IV. At typical operational voltages of STT-MRAM large-contact configurations have *T*_peak_ ranging from 312 K for configuration IV in positive polarity with Si_3_N_4_ passivation to 326 K for configuration III in positive polarity with SiO_2_ passivation.

SiO_2_ passivation provides superior amplification to the self-heating of STT-MRAM with *T*_peak_ that are, on average, 2.6 K higher than comparable simulations for the other considered passivation materials (low-temperature PECVD SiO_2_ and Si_3_N_4_, and Si_3_N_4_). This is observable in Figure 7 and Figure 8.

**Figure 9:**
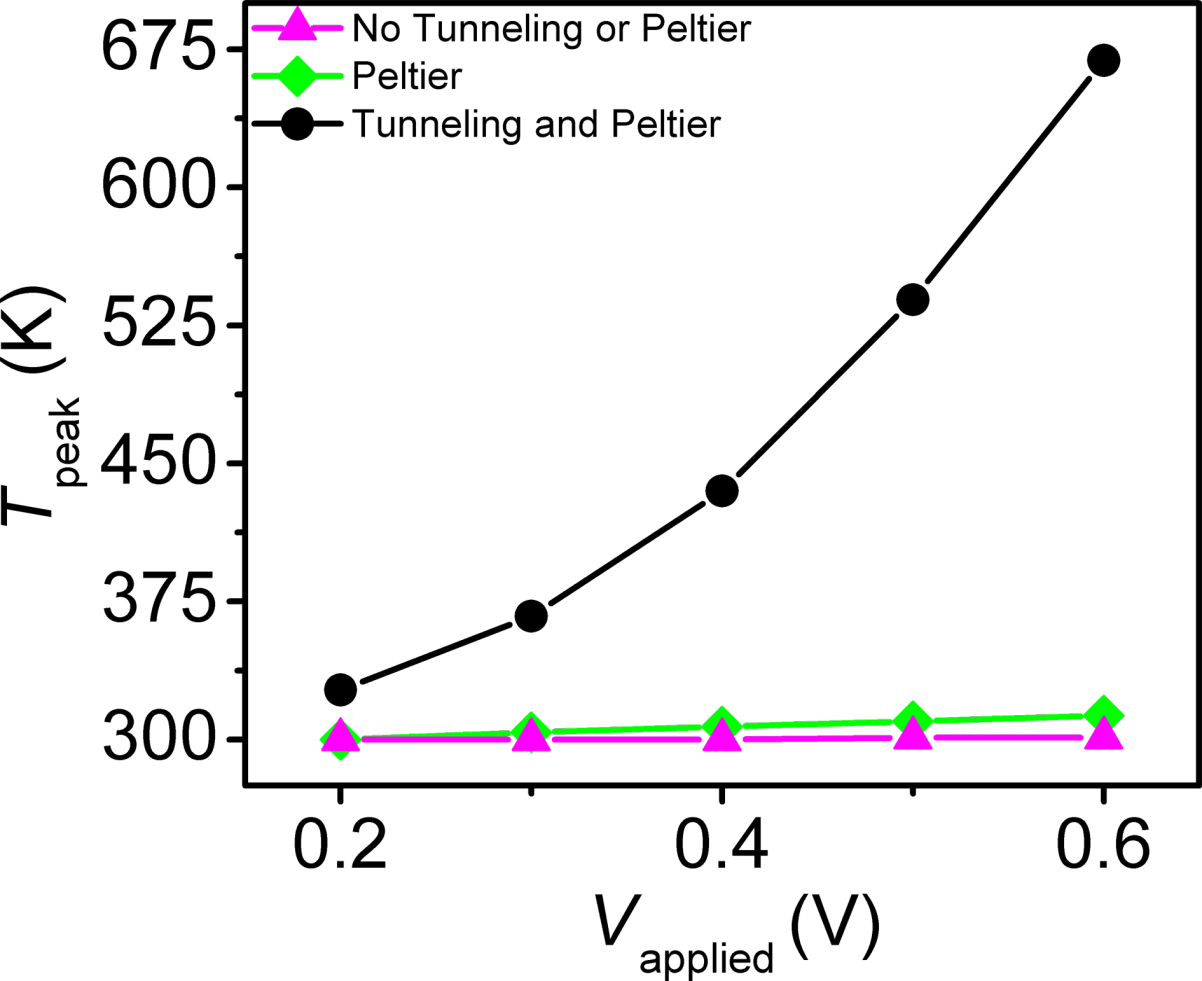
Individual heat contributions from Peltier effect and tunneling around the MTJ for configuration I with SiO_2_ passivation material.

For the extended analysis of SiO_2_ passivation it is observed that small-contact configurations attain peak temperatures, on average, 111.2 K higher than their large-contact counterparts (i.e., the temperatures of configurations I and I-TBR are higher than those of configurations III and III-TBR). This is the result of the passivation material inhibiting heat flow (which is not present in configuration II), and smaller contact regions having lower heat capacity. Additionally, positive polarity yields, on average, 7.9 K higher *T*_peak_ than negative polarity. This is due to reduced current in the negative polarity switching (device is initially in off state) and reduced distance to the thermal boundary for heat generated on the bottom of the MTJ. Assuming constant resistances for the two states – which is equivalent to assuming switching occurs only at the end of the pulses – together with temperature-dependent changes of electrical and thermal conductivity may result in a small underestimation or overestimation of the difference in heating between the positive and negative polarities. Nevertheless, the simulation results appear to be in reasonable agreement with the experimental observations of a reduction by ca. 10% in required power density for the positive polarity reported by Gapihan et al. [20]. Expected temperature asymmetries for opposite current flow under the same resistance state should yield higher temperatures on the lower potential side of the MTJ, similar to the patterns observed in Figures 5–8. However, these cases are not simulated as the thermal effect is mainly of interest for current flow direction and resistance state combinations that result in device switching. Partial inclusion of the different self-heating mechanisms in the simulations shows the relative importance of each (Figure 9). The largest contributor to device heating, by a significant margin, is *Q*_t_. The Peltier and Joule heating in the device are minimal in comparison. As a result, heating is largely localized in the MTJ region as seen in Figure 5 and Figure 6.

Simulations were performed to observe the dependence of thermal behavior on the thickness of the passivation layer and no significant changes were observed between 5 and 20 nm thick layers (except at high voltages and low thermal anchoring where heat saturation occurs).

## Conclusion

In this work, we analyzed the electro-thermal behavior of a standard STT-MRAM device for different passivation layers, current polarity and contact configurations and found that self-heating varies significantly and that, for some cases, it can effectively produce required temperatures of ca. 350 K [2] to about 523 K [3] for thermal assistance at expected operation voltages. The use of high-temperature PECVD SiO_2_ (with slightly higher thermal conductivity and heat capacity compared to low-temperature PECVD SiO_2_) as the passivation material results in significantly higher self-heating compared to the other common passivation materials (Si_3_N_4_ and low-temperature PECVD Si_3_N_4_ and SiO_2_). Positive polarity leads to a 10 K average temperature increase at negative polarity. Interestingly, the results also show that due to tunneling heat, Peltier effect, device geometry, and numerous interfacial layers around the MTJ, the heating on the lower potential side of the junction for a given polarity is higher. This asymmetry is important because thermal assistance requires heating of the free ferromagnetic domain, causing thermal destabilization. But the free domain only experiences significant heating when the device is in positive polarity. Thus care must be taken to ensure the free domain achieves a sufficiently high temperature independent of the overall device temperature.
